# TRPC3-GEF-H1 axis mediates pressure overload-induced cardiac fibrosis

**DOI:** 10.1038/srep39383

**Published:** 2016-12-19

**Authors:** Takuro Numaga-Tomita, Naoyuki Kitajima, Takuya Kuroda, Akiyuki Nishimura, Kei Miyano, Satoshi Yasuda, Koichiro Kuwahara, Yoji Sato, Tomomi Ide, Lutz Birnbaumer, Hideki Sumimoto, Yasuo Mori, Motohiro Nishida

**Affiliations:** 1 Division of Cardiocirculatory Signaling, Okazaki Institute for Integrative Bioscience (National Institute for Physiological Sciences), National Institutes of Natural Sciences, Aichi 444-8787, Japan; 2 Department of Physiological Sciences, SOKENDAI (School of Life Science, The Graduate University for Advanced Studies), Aichi 444-8787, Japan; 3 Department of Translational Pharmaceutical Sciences, Graduate School of Pharmaceutical Sciences, Kyushu University, Fukuoka 812-8582, Japan; 4 Division of Cell-Based Therapeutic Products, National Institute of Health Sciences, Setagaya, Tokyo 158-8501, Japan; 5 Department of Biochemistry, Kyushu University Graduate School of Medical Sciences, Fukuoka 812-8582, Japan; 6 Department of Cardiovascular Medicine Shinshu University School of Medicine, Nagano 390-8621, Japan; 7 Department of Cardiovascular Medicine, Graduate School of Medical Sciences, Kyushu University, Fukuoka 812-8582, Japan; 8 Laboratory of Neuroscience, NIEHS, NIH, Research Triangle Park, NC 27709, USA; 9 Institute for Biomedical Research (BIOMED), Catholic University of Argentina, C1107AFF Buenos Aires, Argentina; 10 Department of Synthetic Chemistry and Biological Chemistry, Graduate School of Engineering, Kyoto University, Kyoto 615-8510, Japan; 11 PRESTO, JST, 4-1-8 Honcho, Kawaguchi, Saitama 332-0012, Japan

## Abstract

Structural cardiac remodeling, accompanying cytoskeletal reorganization of cardiac cells, is a major clinical outcome of diastolic heart failure. A highly local Ca^2+^ influx across the plasma membrane has been suggested to code signals to induce Rho GTPase-mediated fibrosis, but it is obscure how the heart specifically decodes the local Ca^2+^ influx as a cytoskeletal reorganizing signal under the conditions of the rhythmic Ca^2+^ handling required for pump function. We found that an inhibition of transient receptor potential canonical 3 (TRPC3) channel activity exhibited resistance to Rho-mediated maladaptive fibrosis in pressure-overloaded mouse hearts. Proteomic analysis revealed that microtubule-associated Rho guanine nucleotide exchange factor, GEF-H1, participates in TRPC3-mediated RhoA activation induced by mechanical stress in cardiomyocytes and transforming growth factor (TGF) β stimulation in cardiac fibroblasts. We previously revealed that TRPC3 functionally interacts with microtubule-associated NADPH oxidase (Nox) 2, and inhibition of Nox2 attenuated mechanical stretch-induced GEF-H1 activation in cardiomyocytes. Finally, pharmacological TRPC3 inhibition significantly suppressed fibrotic responses in human cardiomyocytes and cardiac fibroblasts. These results strongly suggest that microtubule-localized TRPC3-GEF-H1 axis mediates fibrotic responses commonly in cardiac myocytes and fibroblasts induced by physico-chemical stimulation.

Cardiac fibrosis, characterized by quantitative and qualitative alterations of extracellular matrix (ECM) proteins, is a critical cause of ventricular stiffness as well as impairment of left ventricular (LV) diastolic functions^1^,^2^. There are two types of fibrosis: one is reparative fibrosis, defined as a compensative mechanism to maintain cardiac robustness by replacing the necrotic cardiomyocytes into fibrotic tissue, and the other is responsive fibrosis, defined as an abnormal ECM deposition in the interstitial area of the heart due to hemodynamic overload or inflammation^3^,^4^. Mechanical stress is regarded as the initial stimulus for cardiac remodeling, and several mechano-sensitive or mechano-activated machineries have been suggested to translate changes in physical forces into intracellular signals, including ion channels, sarcomeric proteins, and integrins^5^,^6^,^7^. In addition to these direct sensors of stretch, locally or systemically released humoral factors, such as growth factors and agonists of G protein-coupled receptors, have been implicated in the hypertrophic responses. These signaling pathways together converge on a limited number of intracellular signaling cascades, including Ca^2+^/calmodulin-dependent calcineurin/nuclear factor-activated T cells^8^,^9^, mitogen-activated protein kinases, phosphatidylinositol-3-kinase/Akt, and small GTPases, Ras, Rho, and Rac^10^. Among them, Rho-mediated signaling has been revealed as a critical mediator of fibrosis through actin cytoskeletal reorganization-dependent fibrotic gene transcription^11^,^12^. However, it is still obscure whether responsive Rho-mediated fibrosis can be clearly distinguished from reparative fibrosis during the development of heart failure.

The Rho GTPase activity is fundamentally regulated by its guanine nucleotide exchange factor (GEF), GTPase-activating proteins, and guanine nucleotide dissociation inhibitors, while physical and chemical stimuli primarily stimulate GTP binding to Rho through activating specific RhoGEFs^13^. Among 69 distinct RhoGEF homologues, RhoGEF12 reportedly controls both hypertrophy and fibrosis induced by pressure overload^14^ and A-kinase anchoring protein-Lbc reportedly participates in myofibroblast formation of cardiac fibroblasts induced by angiotensin II or transforming growth factor (TGF)-β^15^. Although several RhoGEFs may participate in the development of cardiac remodeling, the RhoGEF that specifically encodes a signal to induce responsive fibrosis has not been identified.

NADPH oxidase isoform 2 (Nox2) is a microtubule-associated reactive oxygen species (ROS)-producing enzyme that acts as a key mediator of mechanotransductive signaling in normal hearts^16^. Nox2-deficient mice show specific suppression of pressure overload-induced cardiac fibrosis but not hypertrophy^17^. The intracellular Ca^2+^ concentration plays a key role in receptor-stimulated sustained Nox2 activation, and we previously reported that mechanical stress-induced local Ca^2+^ influx through transient receptor potential canonical (TRPC) 3 channel increases Nox2-mediated ROS production in neonatal rat cardiomyocytes (NRCMs)^18^,^19^. TRPC3 forms stable protein complex with Nox2 in myocardial T-tubule, which leads to amplification of ROS signaling in heart^19^. In addition, pharmacological inhibition of TRPC3 actually attenuates LV diastolic dysfunction as well as responsive fibrosis in mouse hearts with dilated cardiomyopathy. However, how the TRPC3-Nox2 axis regulates Rho-mediated responsive fibrosis is unclear. We here demonstrate that TRPC3 deletion specifically inhibits RhoA-mediated maladaptive fibrosis in pressure-overloaded mouse hearts. We also show that a microtubule-associated RhoGEF, GEF-H1, plays a key role in maladaptive fibrosis induced by mechanical stress and TGF-β stimulation.

## Results

### Inhibition of TRPC3 attenuates cardiac fibrosis but not hypertrophy in pressure-overloaded mouse hearts

**Figure 1:**
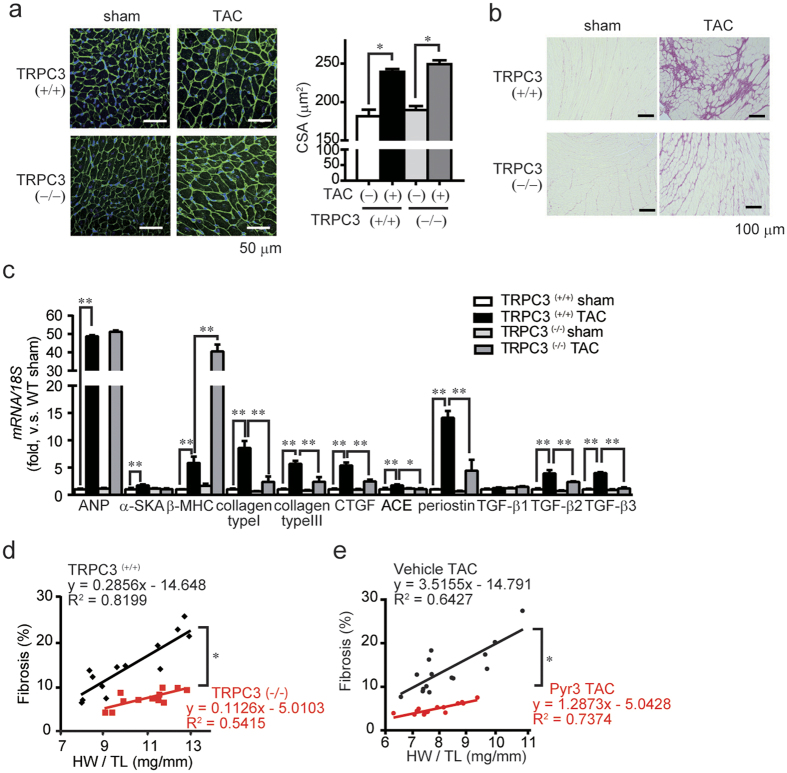
Inhibition of TRPC3 channel activity suppresses cardiac fibrosis but not hypertrophy in pressure overloaded mouse hearts. (**a**) Representative images of wheat germ agglutinin (WGA) staining for cross-sectional areas (CSA) measurement 6 weeks after TAC (left). Green; WGA, blue; DAPI. Quantitative results are shown in right panel. TRPC3^(+/+)^-sham (n = 6), TRPC3^(−/−)^-sham (n = 6), TRPC3^(+/+)^-TAC (n = 13), TRPC3^(−/−)^-TAC (n = 12). (**b**) Representative images of fibrosis 6 weeks after TAC. (**c**) Expression levels of hypertrophy-related and fibrosis-related mRNAs in mouse hearts 1 week after TAC. Sham groups (n = 3), TAC groups (n = 5). (**d,e**) Correlation between fibrosis and hypertrophy 6 weeks after TAC. TRPC3^(+/+)^-TAC (n = 13), TRPC3^(−/−)^-TAC (n = 12), Vehicle-TAC or pyrazole-3 (Pyr3)-TAC (n = 15). Error bars, s.e.m. *P < 0.05, **P < 0.01.

Previous studies using TRPC3-deficient (TRPC3^(−/−)^) mice or a pharmacological inhibitor of TRPC3, pyrazole-3, have revealed that TRPC3 participates in mechanical stress-induced LV diastolic dysfunction in mice^18^,^19^. As interstitial fibrosis is believed as a major cause of LV diastolic dysfunction, we assessed whether TRPC3 inhibition attenuates pressure overload-induced interstitial fibrosis. Although transverse aortic constriction (TAC) significantly increased myocardial cell size in both TRPC3^(+/+)^ and TRPC3^(−/−)^ mice (Fig. 1a), collagen deposition determined by picrosirius red staining demonstrated a marked decrease of fibrosis in TRPC3^(−/−)^ mouse hearts compared to TRPC3^(+/+)^ (Fig. 1b). TAC significantly increased transcription of hypertrophy-related genes, including atrial natriuretic peptide (ANP), α-skeletal muscle actin (α-SKA) and β-myosin heavy chain (β-MHC), in TRPC3^(+/+)^ hearts, and inhibition of TRPC3 never attenuated this induction of hypertrophy-related mRNAs (Fig. 1c and Supplementary Fig. 1a). By contrast, TAC-induced increases in fibrosis-related mRNAs, including those encoding collagen type Iα1 and type IIIα1, CTGF, angiotensin converting enzyme (ACE), periostin, TGF-β2 and TGF-β3, were significantly suppressed by TRPC3 inhibition (Fig. 1c and Supplementary Fig. 1a). The degree of heart weight-to-tibia length ratio (HW/TL) correlated positively with severity of fibrosis in TRPC3^(+/+)^ hearts (Fig. 1d,e). Strikingly, the correlations between HW/TL and fibrosis in TRPC3^(−/−)^ and pyrazole-3-treated hearts indicate that TRPC3 inhibition preferentially suppresses TAC-induced cardiac fibrosis, despite the development of hypertrophy. Lower doses of pyrazole-3 were also found to suppress TAC-induced fibrosis but not hypertrophy (Supplementary Fig. 1b–d). These results strongly suggest that TRPC3 predominantly mediates pressure overload-induced maladaptive fibrosis in mouse hearts.

### GEF-H1 participates in TRPC3-mediated cardiac fibrosis

**Figure 2:**
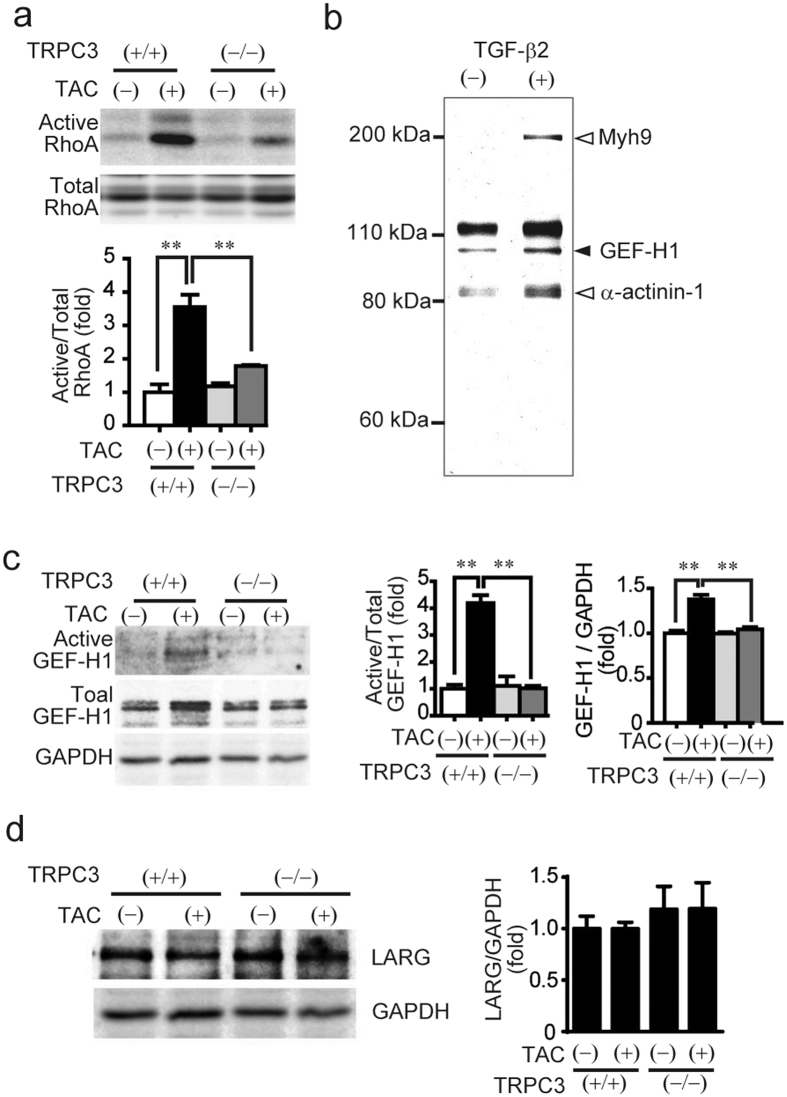
Involvement of GEF-H1 in TRPC3-mediated cardiac fibrosis in pressure overloaded mouse hearts. (**a**) Activation of RhoA in TRPC3^(+/+)^ and TRPC3^(−/−)^ mouse hearts 1 week after TAC (n = 3). (**b**) Representative image of SYPRO Ruby staining of gels loaded with RhoA^G17A^-pulled down proteins. Fibroblasts were stimulated with TGF-β2 (2 ng/mL) for 6 h. (**c**) Activation and expression of GEF-H1 in mouse hearts 1 week after TAC (n = 3). (**d**) Expression of LARG in mouse hearts 1 week after TAC (n = 3). Error bars, s.e.m. *P < 0.05, **P < 0.01.

The small GTP-binding protein RhoA plays a central role in cardiac fibrotic signaling in both cardiomyocytes and cardiac fibroblasts^20^. TAC significantly increased myocardial RhoA activity (Fig. 2a), and the TAC-induced RhoA activation was significantly lower in TRPC3^(−/−)^ than TRPC3^(+/+)^ hearts. Mechanical stretch of cardiomyocytes is well accepted as an appropriate stimulation to initiate profibrotic factors release from cardiomyocytes during pressure overload^21^,^22^, while collagen production from myofibroblasts predominantly determines the severity of fibrosis in heart^22^. As TGF-βs are most prominent profibrotic factors that promote differentiation of cardiac fibroblasts into myofibroblasts and collagen production from myofibroblasts^22^, we examined which RhoGEF(s) can be activated upon TGF-β stimulation in rat cardiac fibroblasts. Pull-down assay using agarose-conjugated nucleotide-free RhoA mutant (RhoA^G17A^) showed that intensities of three bands were significantly increased by TGF-β2 stimulation (Fig. 2b). Among them, only one RhoGEF, GEF-H1, was detected from 110 kDa band by proteomic analysis. RhoGEFs are upstream positive regulators of Rho, and two GEFs, LARG and GEF-H1, are reportedly responsive to mechanical stress^23^. This implies that GEF-H1 acts as a common mediator of pressure overload-induced TRPC3-dependent fibrosis both in cardiomyocytes and cardiac fibroblasts. The level of GEF-H1 protein expression in TAC-operated TRPC3^(+/+)^ heart significantly increased, that was unchanged in TRPC3^(−/−)^ hearts (Fig. 2c). Furthermore, RhoA^G17A^ agarose-dependent pull-down assays revealed that TAC clearly increased binding activity of GEF-H1 in TRPC3^(+/+)^ hearts, and the effect was absent in TRPC3^(−/−)^ hearts (Fig. 2c). In contrast, TAC did not change the level of LARG protein expression (Fig. 2d), suggesting that TAC-induced RhoA activation is mediated predominantly by GEF-H1 in 129 Sv mouse hearts.

### The TRPC3-GEF-H1 axis underlies mechanical stretch-induced RhoA activation and fibrotic gene expressions in NRCMs

**Figure 3:**
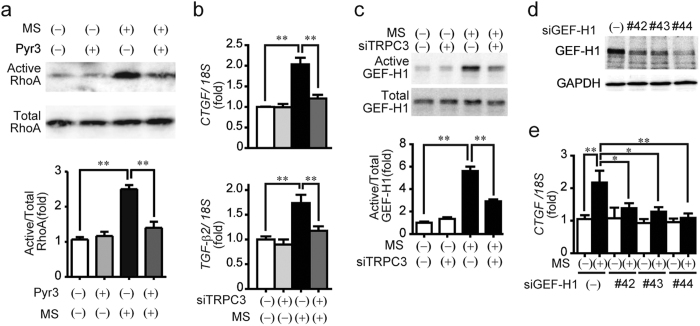
Requirement of TRPC3 in mechanical stress-induced activation of GEF-H1 and RhoA, and induction of fibrosis-related mRNA expressions in NRCMs. (**a**) Effect of pyrazole-3 (Pyr3) on RhoA activation induced by mechanical stretch (MS) in NRCMs. NRCMs were treated with Pyr3 (1 μM) 20 min before MS (20% static-MS for 10 min). Lower panel is the quantitative result of RhoA pulldown assay (n = 3). (**b**) Effect of TRPC3 knockdown on MS-induced increase in fibrosis-related genes (CTGF and TGF-β2). NRCMs were subjected to 20% static-MS for 6 h. (**c**) Effects of TRPC3 knockdown on GEF-H1 activation. NRCMs were subjected to 20% static-MS for 10 min (n = 3). (**d,e**) Effects of GEF-H1 siRNAs (#42, 43 and 44) on GEF-H1 protein (**d**) and CTGF mRNA (**e**) expressions induced by MS (20% static MS for 6 h) in NRCMs (n = 4). Error bars, s.e.m., *P < 0.05, **P < 0.01.

Excess LV diastolic filling due to chronic pressure overload is believed as a major cause of cardiac fibrosis *in vivo*, and mechanical stretch of cardiomyocytes is accepted as an appropriate *in vitro* model to mimic chronic pressure overload in heart^22^. We have also reported that mechanical stretch-induced ATP/UDP release from cardiomyocytes through pannexin-1 channels triggers pressure overload-induced cardiac fibrosis in *in vivo* mouse hearts^21^. We next examined whether a TRPC3-GEF-H1 axis underlies mechanical stretch-induced RhoA activation and fibrotic gene expressions in NRCMs. Mechanical stretch of NRCMs increased RhoA activity and expression of two RhoA-dependent mRNAs, connective tissue growth factor (CTGF) and TGF-β2, and these RhoA-dependent responses were suppressed by TRPC3 inhibition (Fig. 3a,b). Consistent with the results of TAC-operated heart, mechanical stretch-activated GEF-H1 was significantly suppressed in TRPC3-silencing NRCMs^19^ (Fig. 3c). In addition, GEF-H1-silencing NRCMs also suppressed mechanical stretch-induced increases in CTGF mRNA (Fig. 3d,e). These results suggest that the TRPC3-GEF-H1 axis underlies Rho-dependent fibrotic responses of cardiomyocytes induced by mechanical stretch.

### Involvement of the TRPC3-GEF-H1 axis in TGF-β2-induced fibrotic response in rat cardiac fibroblasts

**Figure 4:**
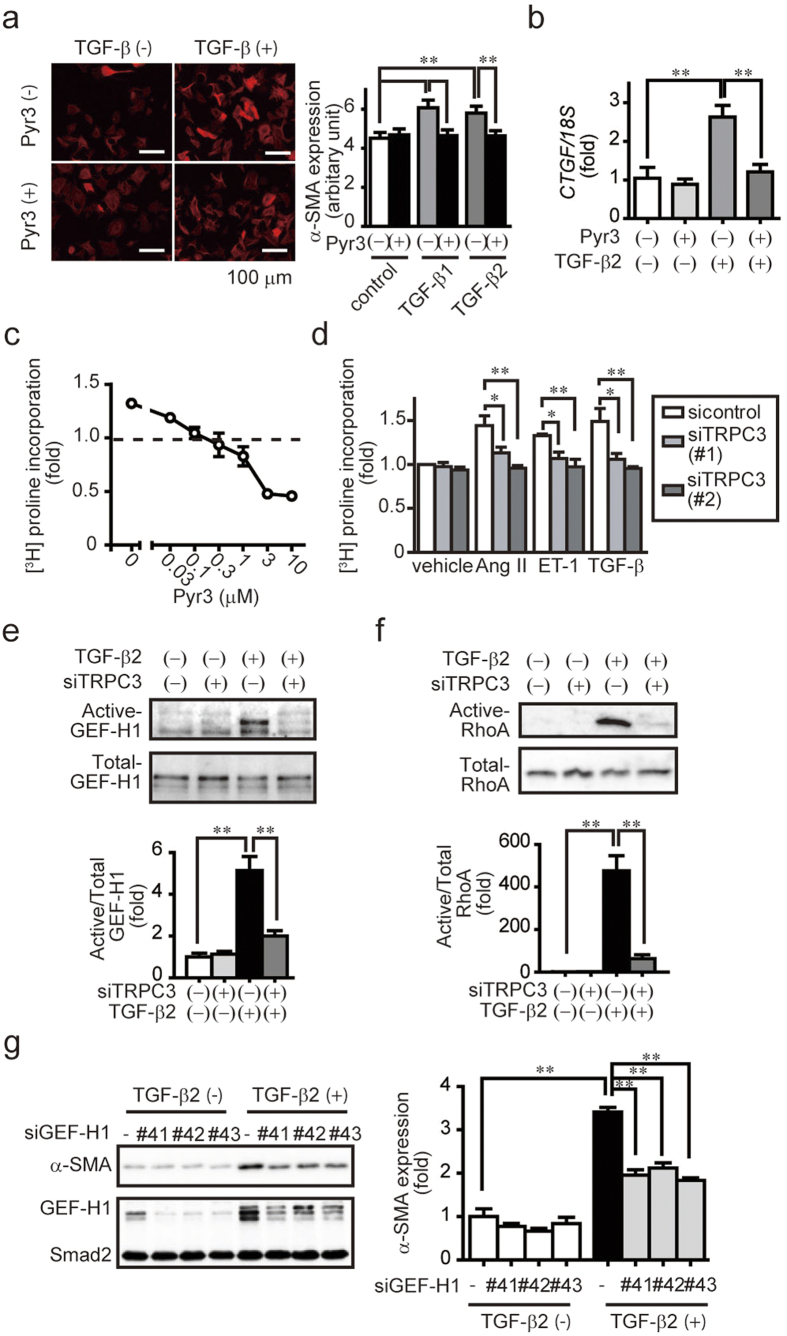
Inhibition of TRPC3 attenuates TGF-β-induced myofibroblast transdifferentiation of neonatal rat cardiac fibroblasts. (**a**) Representative α-SMA-stained images of rat cardiac fibroblasts. Cells were treated with Pyr3 (1 μM) for 20 min prior to treatment with TGF-β2 (2 ng/mL) for 48 h (n = 3). (**b,c**) Effect of Pyr3 (1 μM) on TGF-β2 (2 ng/mL for 6 h)-induced CTGF mRNA expression (**b**) and [^3^H] proline incorporation (**c**) in fibroblasts (n = 3). (**d**) Effects of TRPC3 knockdown on [^3^H] proline incorporation in fibroblasts with or without Ang II (100 nM), endothelin-1 (ET-1; 100 nM) or TGF-β2 (n = 3). (**e,f**) Effects of TRPC3 knockdown on GEF-H1 (**e**) and RhoA (**f**) activation induced by TGF-β2 for 6 h. (n = 3) (**g**) Effects of GEF-H1 knockdown on the TGF-β2-induced increase in α-SMA expression (n = 3). Error bars, s.e.m. *P < 0.05, **P < 0.01.

Although profibrotic factors released from cardiomyocytes may trigger pressure overload-induced cardiac fibrosis, myofibroblast differentiation and collagen production from cardiac fibroblast must be a major cause for the progression of fibrosis driving maladaptive cardiac remodeling^22^,^24^. We therefore asked whether the TRPC3-GEF-H1 axis participates in TGF-β-induced transdifferentiation of cardiac fibroblasts into myofibroblasts. Treatment with pyrazole-3 significantly suppressed TGF-β2-induced α-smooth muscle actin expression, an index of myofibroblast formation, CTGF mRNA induction, and [^3^H]proline incorporation, an index of collagen synthesis^25^ (Fig. 4a–c). TRPC3 knockdown suppressed [^3^H]proline incorporation induced by fibrogenic ligands such as angiotensin II, endothelin-1 and TGF-β2 (Fig. 4d). TRPC3 knockdown also suppressed TGF-β2-induced activation of GEF-H1 and RhoA (Fig. 4e,f), and GEF-H1 knockdown suppressed TGF-β2-induced myofibroblast formation (Fig. 4g). These results strongly suggest that TRPC3 also participates in TGF-β-induced fibrotic responses of cardiac fibroblasts through activation of GEF-H1/RhoA-dependent signaling pathways.

### Involvement of microtubule-associated NADPH oxidase in TRPC3-mediated GEF-H1 activation

**Figure 5:**
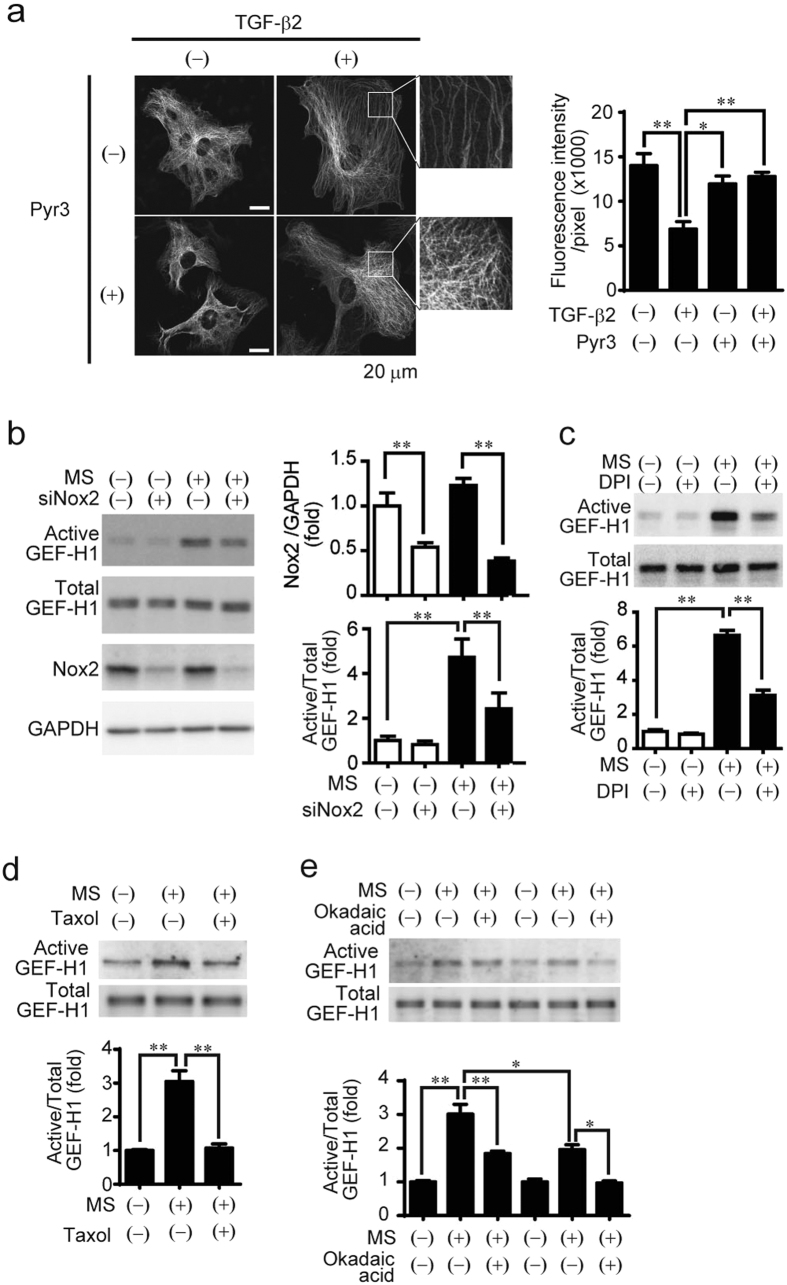
Involvement of microtubule stability in TRPC3/Nox2-mediated GEF-H1 activation. (**a**) Representative images of microtubule organization in rat cardiac fibroblasts with or without TGF-β2 (2 ng/mL) for 6 h, in the presence or absence of Pyr3 (1 μM). Right panels were magnified views indicated with white boxes. Bar graph represents the semi-quantitative results of microtubule densities (n = 6). (**b,c**) Effects of knockdown (**b**) or pharmacological inhibition (**c**) of Nox2 on GEF-H1 activation induced by MS. NRCMs were subjected to 20% static-MS for 10 min (n = 3). (**d**) Involvement of tubulin stability on MS-induced GEF-H1 activation in NRCMs. NRCMs were treated with taxol (1 μM) 30 min prior to 20% static-MS for 10 min (n = 3). (**e**) Effect of Nox2 knockdown on MS-induced GEF-H1 activation in NRCMs. NRCMs were treated with okadaic acid (0.1 μM) 30 min prior to 20% static-MS for 10 min (n = 3). Error bars, s.e.m. *P < 0.05, **P < 0.01.

We further asked how TRPC3-mediated Ca^2+^ influx specifically activates GEF-H1 in cardiac cells. GEF-H1 is predominantly localized in microtubules, and both microtubule-dependent and –independent activation mechanism are involved in ligand-stimulated GEF-H1 activation^26^. Our previous study suggested a functional coupling between TRPC3 and microtubule-associated Nox2, and that TRPC3/Nox2-mediated production of reactive oxygen species (ROS) is highly correlated with the severity of heart failure^19^. Microtubule-associated GEF-H1 is reportedly activated by ROS through dissociation from tubulin^27^, and we previously reported that TRPC3 mediates mechanical stretch-induced Nox2 activation in NRCMs^19^. Therefore, we tested whether the TRPC3-Nox2 signaling pathway contributed to GEF-H1 activation. A pretty polymerized tubulin was observed in normal rat cardiac fibroblasts (Fig. 5a). TGF-β2 stimulation significantly reduced the density of microtubule, which was canceled by TRPC3 inhibition. This result was correlated well with that of GEF-H1 activity (Fig. 4e). Inhibition of Nox2 by the treatment with Nox2 siRNA or diphenyleneiodonium (DPI) significantly suppressed the mechanical stretch-induced GEF-H1 activation in NRCMs (Fig. 5b,c). Despite almost complete knockdown of Nox2 protein by Nox2 siRNA, partial GEF-H1 activation was still observed in Nox2-silencing NRCMs. In contrast, mechanical stretch-induced GEF-H1 activation was abolished completely in NRCMs pretreated with taxol, a microtubule stabilizing agent (Fig. 5d). This indicates that mechanical-stretch induced GEF-H1 activation mainly depends on microtubule depolymerization in cardiomyocytes. As the residual GEF-H1 activation in Nox2-silencing NRCMs was completely abolished by okadaic acid, an inhibitor of PP2A phosphatase, which is reported to positively regulates GEH-H1 activity in a microtubule-independent manner^26^ (Fig. 5e). These results suggest that TRPC3 participates in mechanical stress-induced GEH-H1 activation, in part through Nox2/ROS-mediated microtubule depolymerization.

### Inhibition of TRPC3 attenuates fibrotic responses of human cardiomyocytes and cardiac fibroblasts

**Figure 6:**
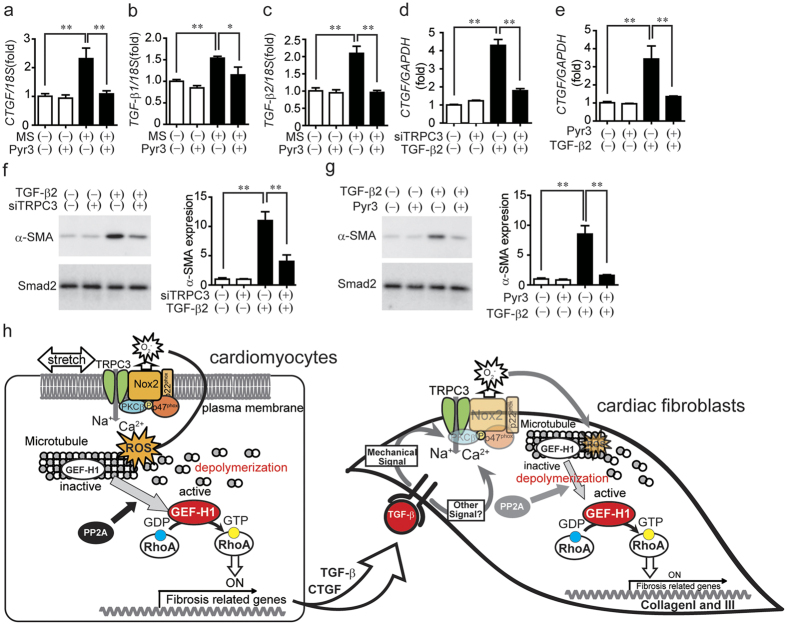
Inhibition of TRPC3 attenuates fibrotic response in human iPSC-derived cardiomyocytes or human cardiac fibroblasts. (**a–c**) Effect of Pyr3 (1 μM) on induction of fibrosis-related mRNAs (CTGF and TGF-β1 or -β2) by MS in human iPSC-derived cardiomyocytes (n = 3). Cells were subjected to 20% static-MS for 3 h. GAPDH and 18 S ribosomal RNA were used as internal controls. (**d–g**) Effects of knockdown (**d**) or pharmacological inhibition (**e**) of TRPC3 on CTGF mRNA expression (**d,e**) and α-SMA protein expression (**f,g**) induced by TGF-β2 stimulation in human cardiac fibroblasts (n = 3). Error bars, s.e.m. *P < 0.05, **P < 0.01. (**h**) Schema for the mechanism of TRPC3-mediated cardiac fibrosis induced by pressure overload. TRPC3 mediates MS-induced expression of profibrotic factors (CTGF and TGF-β) in cardiomyocytes and TGF-β-induced fibrotic responses of cardiac fibroblasts, partially through Nox2-dependent GEF-H1 activation.

Finally, we used human induced pluripotent stem cell (iPSC)-derived cardiomyocytes and human cardiac fibroblasts to investigate whether TRPC3-induced fibrogenic signaling is conserved across species. Mechanical stretch of human iPSC cardiomyocytes increased expression of CTGF, TGF-β1 and TGF-β2 mRNAs, which were significantly suppressed by TRPC3 inhibition (Fig. 6a–c). Stimulation of human cardiac fibroblasts with TGF-β2 increased the levels of CTGF mRNA expression and α-SMA protein expression; both were suppressed by TRPC3 inhibition (Fig. 6d–g). These results suggest that TRPC3/Nox2 communication may be a common mechanism underlying activation of RhoA-dependent fibrogenic signaling induced by mechanical stretch and TGF-β stimulation in human cardiac cells.

## Discussion

The roles of cytoskeletal alterations especially of microtubules, formed by polymerization of α- and β-tubulin, and desmin have been implicated in the development of cardiac hypertrophy and heart failure in numerous experimental studies. The contractile myofilaments are reportedly reduced during the development of cardiac remodeling, while cytoskeletal proteins including microtubules are compensatively disorganized, depolymerized, and increased in amount thereby posing an increased load on myocytes which impedes sarcomere motion and promotes cardiac dysfunction. Although the molecular mechanism underlying microtubule destabilization-mediated cardiac fibrosis has been precisely unclear, the present study demonstrated that GEF-H1 mediates Rho-dependent fibrotic responses of cardiomyocytes induced by mechanical stress and cardiac fibroblasts induced by TGF-β stimulation. Although TGF-β-induced epithelial-to-mesenchymal transition of normal murine mammary gland (NMuMG) epithelial cells results in decreased stiffness and loss of normal stiffening response to force applied on integrins through TGF-β/ALK5-enhanced proteasomal degradation of LARG and GEF-H1^28^, GEF-H1 protein expression levels were not reduced in pressure-overloaded mouse hearts and TGF-β-stimulated rat cardiac fibroblasts. We cannot explain the difference of GEF-H1 stability after TGF-β stimulation between NMuMG cells and cardiac cells, but the maintenance of GEF-H1 stability can explain the mechanism why TGF-β preferentially induces cardiac fibrosis as well as cardiac stiffness.

We have identified how TRPC3-mediated local Ca^2+^ influx specifically encodes signals to induce maladaptive fibrosis. TRPC3 contributes to the transition from adaptive hypertrophy to maladaptive hypertrophy, including fibrosis, induced by pressure overload in mice (Fig. 1). The inhibition of TRPC3 suppressed GEF-H1 activation induced by mechanical stretch in cardiomyocytes (Fig. 3) and TGF-β stimulation in rat cardiac fibroblasts (Fig. 4). Specifically, the TRPC3-Nox2 communication mediates mechanical stress-induced fibrosis through activation of a GEF-H1-RhoA signaling axis (Fig. 5). GEF-H1 is reportedly activated by ROS-mediated microtubule depolymerization^27^, which establishes the specificity of TRPC3/Nox2-derived ROS signaling in which microtubules are a critical component^16^. Mechanical stretch-induced GEF-H1 activation was abolished by microtubule stabilization, suggesting that microtubule-dependent GEF-H1 regulation is predominant. However, Nox2 downregulation significantly suppressed GEF-H1 activity but not completely. Recently, G_α_ and G_β_ subunits of trimeric G protein and PP2A phosphatase were identified as other positive regulators for GEF-H1 activation^26^. The residual activity of GEF-H1 was indeed sensitive to PP2A inhibitor, okadaic acid (Fig. 5). These results suggest that GEF-H1 activation by mechanical stretch is not solely regulated by microtubule, but other pathways such as dephosphorylation by PP2A are also important. It is unlikely that mechanical stretch activates all the GEF-H1 expressed in NRCMs. The small pool of GEF-H1 activated by mechanical stretch would be localized presumably near TRPC3-Nox2 complex. This is consistent well with our concept which TRPC3-mediated local Ca^2+^ influx specifically encodes signals to induce maladaptive fibrosis via close coupling with Nox2.

Another novel finding is to demonstrate that TRPC3 can be downstream mediator of TGF-β stimulation in cardiac fibroblasts. Our aim is to investigate whether TRPC3-GEF-H1 axis can act as a common mediator of fibrotic signaling both in cardiomyocytes and cardiac fibroblasts, while there were no evidence supporting a functional relationship between TRPC3 and TGF-βs. TRPC3 was originally identified as a molecular candidate of receptor-activated cation channels that can be activated by inositol 1,4,5-trisphosphate (IP_3_) and 1,2-diacylglycerol (DAG) produced by phophatidylinositol-dependent phospholipase C (PI-PLC)^29^, and we have previously reported that TRPC3 can be also activated by mechanical stretch in NRCMs^18^,^19^. However, TGF-β stimulation seems not to activate PI-PLC in cardiac fibroblasts, because we have never observed a transient increase in intracellular Ca^2+^ concentration caused by IP_3_-mediated Ca^2+^ release from endoplasmic reticulum using whole cell Ca^2+^ imaging. In contrast, TGF-β stimulation actually increased the number of Ca^2+^ spike frequency (i.e., spontaneous activity) in NRCMs, which was completely suppressed by pyrazole-3 (Supplementary Fig. 2). This strongly suggests that TGF-β stimulation actually induces cation influx through TRPC3 channel, leading to increase in plasma membrane potential (i.e., depolarization)^9^. Although our results indirectly suggested a requirement of TRPC3-mediated Ca^2+^ influx in TGF-β-stimulated ROS production and GEF-H1 activation through Nox2, future work focusing on microtubule-localized Ca^2+^ signaling will be required to elucidate the molecular mechanism underlying activation of TRPC3 by TGF-β stimulation.

Fibrotic cardiac disease, emerging as loss of LV function following maladaptive tissue remodeling, is a leading cause of death worldwide. The pharmacological inhibition of TRPC3 attenuates mechanical stretch-induced fibrotic responses in human iPSC-derived cardiomyocytes and cardiac fibroblasts (Fig. 6). Since TRPC3 is ubiquitously expressed not only in the heart but also in blood vessels, kidney, liver and lung, TRPC3 could be a novel therapeutic target for the treatment of human fibrotic diseases.

## Methods

### Animals

All protocols using mice and rats were reviewed and approved by the ethics committees at the National Institutes of Natural Sciences or the Animal Care and Use Committee, Kyushu University, and were performed according to the institutional guidelines concerning the care and handling of experimental animals. 129 Sv mice with homozygous deletion of the gene encoding TRPC3 were provided by the Comparative Medicine Branch, National Institute of Environmental Health Sciences, Research Triangle Park, North Carolina 27709. Genotyping was performed using PCR primers; TRPC3-A 5′-GAATCCACCTGCTTACAACCATGTG-3′ and TRPC3-B 5′-GGTGGAGGTAACACACAGCTAAGCC-3′. The PCR was performed using Phusion High-Fidelity DNA polymerase (Thermo Scientific). Mice were maintained in specific-pathogen-free area under a 12 h/12 h light/dark cycle. C57BL/6J mice were purchased from SLC. Sprague-Dawley rats were purchased from Kyudo or SLC.

### Pressure overload study in mice

Pressure overload by TAC was performed as described previously^19^,^30^. Briefly, male mice (6–8 weeks old) were anaesthetized using a mixture of domitor (Zenoaq), midazolam (Sando) and butorphanol (Meiji Seika Pharma). After orotracheal intubation and ventilation, an intercostal space was opened. The transverse aorta was then exposed and constricted between the brachiocephalic artery and left carotid artery to the width of a 27-G needle using a 5-0 silk braid. Sham treatment was performed similarly but without constriction of the silk braid. An osmotic minipump (model 2004 (Alzet)) filled with vehicle (polyethylene glycol) or pyrazole-3 (10, 30 or 100 μg kg^−1^ day^−1^) was implanted intraperitoneally^19^,^30^.

### Morphological analysis

The LV functions of mice 6-week after TAC were assessed using a micronanometer catheter, and results of hemodynamic parameters were reported elsewhere^19^,^30^. Hearts were removed after LV pressure measurement, washed in PBS and fixed in 10% neutral buffered formalin. For quantitative assessment of collagen type I and III deposition, the hearts were embedded in paraffin, sectioned at a thickness of 3 μm, and stained with picrosirius red using 0.1% Direct Red 80. To assess CSA of cardiomyocytes, the sections were stained with Alexa Fluor 488-conjugated wheat germ agglutinin (WGA) (Life technologies). Three regions were selected at random for each left ventricle, and the average values were calculated using a BZ-II Analyzer (Keyence).

### Isolation of cardiomyocytes and cardiac fibroblasts from neonatal rats

Rat pups were sacrificed on postnatal day 1–3, after which the left ventricles were removed and minced. The minced tissue was pre-digested in 0.05% trypsin-EDTA (Gibco) over night at 4 °C and then digested in 1 mg ml^−1^ collagenase type 2 (Worthington) in PBS for 30 min at 37 °C. The dissociated cells were plated in a 10-cm culture dish and incubated at 37 °C in a humidified atmosphere (5% CO_2_, 95% air) for 1 hour in DMEM containing 10% FBS and 1% penicillin and streptomycin. Attached cells were cardiac fibroblast and cultured in same medium. Floating cells were collected and plated into gelatin-coated culture dishes or laminin-coated stretch chamber dishes at a density of around 1.5 × 10^5^ cells/cm^2^. After 24 h, the culture medium was changed to serum-free DMEM. For protein knockdown, cells were transfected with siRNAs (100 nM) using Lipofectamine 2000 for 72 h. Primary human cardiac fibroblasts were purchased from Lonza and cultured according to manufacturer’s instruction.

### iPSC-derived cardiomyocytes

The 253G1 human iPSC line was provided by the RIKEN BRC through the Project for Realization of Regenerative Medicine and the National Bio-Resource Project of the MEXT, Japan^31^. Differentiation of human iPSCs into cardiomyocytes was performed using the previously described protocol^32^. The iPSCs were cultured in mTeSR1 medium (STEMCELL Technologies) on Matrigel (BD Biosciences)-coated dishes, and a ROCK inhibitor (Y-27632; Wako, 10 μM) was added to the cultured medium for 1 h. The iPSCs were then dissociated into single cells using Accutase (Life technologies) for 10 min at 37 °C, seeded onto Matrigel-coated dishes at density of 10^5^ cells/cm^2^, and incubated for 24 h in mTeSR1 medium supplemented with 10 μM Y-27632. The medium was then changed to mTeSR1 without Y-27632 and refreshed daily for 4 days. Thereafter, on day 0 the cells were treated with 12 μM GSK3 inhibitor (CHIR99021; STEMGENT) in RPMI/B27-insulin medium for 24 h. The medium was then changed, and the cells were incubated in RPMI/B27-insulin for an additional 48 h (days 1–2). The cells were then treated with 5 μM inhibitor of Wnt production-4 (IWP4; STEMGENT) in RPMI/B27-insulin for 48 h (days 3–4), after which the medium was changed to RPMI/B27-insulin for 48 h (day 5–6). Beginning on day 7, the cells were maintained in RPMI/B27, and the medium was changed every 3 days. On day 20, the cells were dissociated into single cells using Accutase for 30 min at 37 °C and seeded onto human Laminin-211 (BioLamina)-coated stretch chamber dishes (Menicon) at a density of 10^6^ cells/dish in RPMI/B27 supplemented with 10 μM Y-27632. After 24 h, the medium was replaced with fresh RPMI for 24 h, and the cells were used for experimentation.

### Measuring mRNA expression in cells and tissues

Total RNA was isolated from frozen mouse heart samples using an RNeasy Fibrous Tissue Mini Kit (Qiagen) or from cardiac cells using an RNeasy Mini Kit (Qiagen) according to the manufacturer’s instructions. Quantitative real-time PCR was performed using an ABI PRISM 7500 Real-Time PCR System (Applied Biosystems) and a OneStep RT-PCR Kit (Qiagen) according to the manufacturer’s instructions. All Taqman probes used were purchased from Applied Biosystems.

### GST-RBD and RhoA^G17A^-agarose pull-down

To purify the recombinant Rho-binding domain (RBD) of Rhotekin tagged with glutathione S-transferase (GST) proteins, *Escherichia coli* cells transfected with pGEX-4T1 harboring Rhotekin-RBD were incubated with 100 μM isopronyl β-D-1-thioglactopyranoside (IPTG) for 24 h at 25 °C. *E. Coli* were then sonicated and lysed in lysis buffer containing 20 mM HEPES (pH 7.4), 150 mM NaCl, 5 mM MgCl_2_, 1% (v/v) triton X-100, 1 mM dithiothreitol (DTT), 0.2 μM phenylmethylsulfonyl fluoride, 0.2 μg ml^−1^ aprotinin and 0.2 μg ml^−1^ leupeptin, and the GST-RBD was purified from the lysate by incubation with glutathione-Sepharose 4B beads (GE Healthcare) at 4 °C for 1 h.

Active RhoA-pull-down experiments were carried out as described previously^21^. Hearts and cardiac cells were homogenized and lysed in RBD buffer containing 50 mM Tris-HCl (pH 7.6), 500 mM NaCl, 10 mM MgCl_2_, 1% (v/v) Triton X-100, 0.1% (w/v) SDS, 0.5% (w/v) deoxycholate, 50 mM NaF, 2 mM Na_3_VO_4_ and a protease inhibitor cocktail (Nacalai). The resultant lysates were clarified by centrifugation at 15,000 r.p.m. for 10 min at 4 °C, and the supernatants were equalized for total volume (700 μl) and the amount of protein (1 mg). The samples were then rotated for 30 min with 30 μg of purified GST-RBD bound to glutathione-Sepharose 4B beads, after which the beads were washed twice in RBD wash buffer containing 50 mM Tris-HCl (pH 7.6), 500 mM NaCl, 10 mM MgCl_2_ and 1% (v/v) Triton X-100; suspended in SDS sample buffer; and boiled for 5 min.

RhoGEF pull-down assays were performed as described previously with the following modification^23^. Hearts and cardiac cells were homogenized and lysed in RhoGEF buffer containing 20 mM HEPES (pH 7.5), 150 mM NaCl, 1% (v/v) Triton X-100, 5 mM MgCl_2_, 1 mM DTT, and a protease inhibitor cocktail (Nacalai). After clarifying the lysates by centrifugation at 15,000 r.p.m. for 10 min at 4 °C, the supernatants were equalized for total volume (1 ml) and the amount of protein (1 mg), and samples were rotated for 30 min with a 40-μl bed volume of RhoA^G17A^ Agarose Beads (Cell Biolabs). The beads were then washed twice by RhoGEF buffer, suspended in SDS sample buffer and boiled for 5 min. Acrylamide gels were stained with SYPRO Ruby protein gel stain (Thermo) according to manufacturer’s instruction. Excised gels were dehydrated by acetonitrile, and proteins were reduced by DTT, alkylated by iodoacetamide and digested by trypsin (Promega). Peptides were analyzed with liquid chromatography–mass spectrometry (EASY-nLC1000 and Orbitrap Elite, Thermo). Data were analyzed by Mascot ver.2.5.1 (Matrix science).

### Immunohistochemical staining

Neonatal rat cardiac fibroblasts were fixed in 4% paraformaldehyde in PBS right after TGF-β2 treatment. The fixed cells were permeabilized using 0.1% Triton X-100 in PBS for 5 min on ice then blocked using 1% FBS in PBS for 1 h at room temperature. Anti-α-SMA (Sigma) antibodies were diluted in incubation solution containing 0.01% (v/v) Triton X-100, 5% (w/v) BSA and 3% (v/v) FBS in PBS over night at 4 °C. After incubation with the secondary antibody and nuclear staining using DAPI, images were captured using a confocal laser-scanning microscope (FV-10i, Olympus).

### Confocal imaging and semi-quantitative analysis of tubulin depolymerization

Rat cardiac fibroblasts were transfected with mEmerald-fused tubulin by electroporation (1200 V, 10 msec, and 2 pulses) by Neon transfection system (Life technologies) and plated onto fibronectin-coated glass-base dishes (Iwaki). 48 h after transfection, cells were serum starved for 24 h and stimulated with TGF-β2 (2 ng/mL) for 6 h at 37 °C in the presence or absence of Pyr3 (1 μM). Cells were fixed with ice-cold methanol followed by rehydration with PBS. Fluorescence images were acquired with a confocal laser-scanning microscope (A1Rsi; Nikon) using the 488 nm line of an argon laser for excitation and a 493 nm to 552 nm band-pass filter for emission for mEmerald. The specimens were viewed using plan oil objectives (PlanApo-VC 100xH, numerical aperture 1.4). Microtubule depolymerization was quantified by measuring the density of microtubule using imageJ software. Mean fluorescence intensity within 3 region of interest (200 × 200 pixels) per cells were calculated and represented as normalized fluorescence intensity per pixel. ROIs were placed avoiding microtubule-dense area around nucleus. Values from 6 cells per condition from 3 independent experiments were averaged.

### [^3^H] proline incorporation assay

Rat cardiac fibroblasts were treated with [^3^H] proline 2 h after agonist stimulation and incubated for further 6 h. Cells were washed with ice-cold PBS and fixed in 10% trichloroacetic acid. Cells were lysed in 1 N NaOH, and incorporated [^3^H] proline was measured by a liquid scintillation counter.

### Statistical Analysis

Results are presented as the mean ± s.e.m. from at least three independent experiments. Statistical comparisons were made using Student’s *t*-test (for two groups) or analysis of variance followed by the Student-Newman-Keuls procedure (for multiple groups). Values of P < 0.05 were considered significant.

## Additional Information

**How to cite this article:** Numaga-Tomita, T. *et al*. TRPC3-GEF-H1 axis mediates pressure overload-induced cardiac fibrosis. *Sci. Rep.* **6**, 39383; doi: 10.1038/srep39383 (2016).

**Publisher's note:** Springer Nature remains neutral with regard to jurisdictional claims in published maps and institutional affiliations.

## Supplementary Material

Supplementary Figures
