# Supplementary material for: TRPC3-GEF-H1 axis mediates pressure overload-induced cardiac fibrosis
Source: Sci Rep. 2016 Dec 19;6:39383. doi: 10.1038/srep39383 (PMC5171702; doi:10.1038/srep39383)
Supplement: Supplementary Figures [file srep39383-s1.pdf]

TRPC3-GEF-H1 axis mediates pressure overload-induced cardiac fibrosis

Takuro Numaga-Tomita, Naoyuki Kitajima, Takuya Kuroda,

Akiyuki Nishimura, Kei Miyano, Satoshi Yasuda, Koichiro Kuwahara, Yoji Sato,

Tomomi Ide, Lutz Birnbaumer, Hideki Sumimoto, Yasuo Mori

and Motohiro Nishida

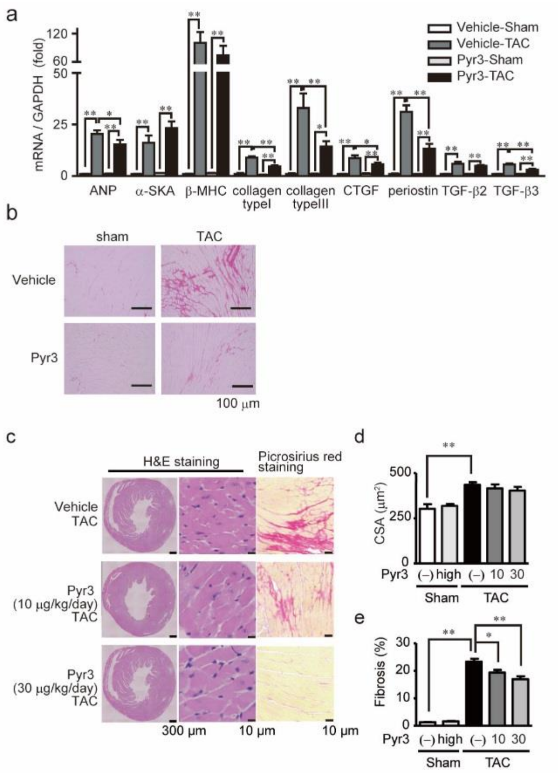

Supplementary Figure 1 : Pyrazole-3 suppresses pressure overload-induced LV fibrosis in C57BL6/J mice.

(**a**) mRNAs of hypertrophy-related and fibrosis-related genes in mice hearts 1 week after TAC (n=4). Pyrazole-3 (Pyr3) or vehicle was administrated 1 week before TAC. (**b**) Representative images of picrosirius red staining 6 weeks after TAC. (**c**) Representative images of H&E staining and picrosirius red staining 6 week after TAC. Pyr3 (10 or 30 μg/kg/day) or vehicle was administrated 1 week after TAC. (**d**) Cross-sectional area (CSA) of cardiomyocytes 6 weeks after TAC (n=4). (**e**) Results of interstitial fibrosis 6 weeks after TAC with or without Pyr3 (n=4). Error bars, s.e.m. ∗P<0.05, ∗∗P<0.01.

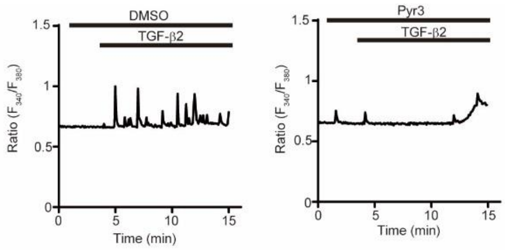

Supplementary Figure 2 : Pyrazole-3 suppresses TGF-β-induced Ca^{2+} oscillations in NRCM. Time courses of intracellular Ca^{2+} mobilization in NRCM stimulated with 10 ng/mL TGF-β2. Cells were treated with 1 μM Pyr3.
